# Supplementary material for: Content-rich biological network constructed by mining PubMed abstracts
Source: BMC Bioinformatics. 2004 Oct 8;5:147. doi: 10.1186/1471-2105-5-147 (PMC528731; doi:10.1186/1471-2105-5-147)
Supplement: Additional File 5 — The original Chilibot query results of the term "long-term potentiation (LTP)" and 22 other terms, limiting the latest references analyzed to the years 1990, 1995, 2000, and 2004. [file 1471-2105-5-147-S5.bz2 › chilibotAdditionalFile5/ltp2004/html/KV4.2.html]

 


**KV4.2** (Input: KV4.2 ) 

---


**Google Searches:** Entire Web  | EDU domain only  | PDF files only

.

**External Links:** OMIM |
LocusLink
| Swissprot
| GeneCards

  
**Maps of KV4.2**

Simple
Complete
graph in
radiant
tree
square
layout.

**New Hypothesis !**

**Synonyms** 

- kv4.2   [PubMed]

**Synopsis**

- These results suggest that **Kv4.2** is probably a major isoform contributing to I A in hippocampus neurons.  Sheng Li Xue Bao, 2003 
  [23]

- Simulations with an allosteric model of inactivation, supported by the experimental data, suggested that, in response to membrane depolarization, **Kv4.2** channels accumulate in the closed inactivated state s, from which they directly recover, bypassing the open state.  J Physiol, 2001 
  [20]

- The functional properties of **Kv4.2**, especially the flecainide sensitivity, resemble those of ITO in rat and human myocytes better than those of Kv1.4.  Cardiovasc Res, 1997 
  [19]

- These observations indicate that **Kv4.2** is a substrate for ERK in vitro and in vivo, and suggest that ERK may regulate potassium channel function by direct phosphorylation of the pore forming alpha subunit.  J Neurochem, 2000 
  [16]

- These results provide insight into likely sites of regulation of **Kv4.2** by PKA.  J Biol Chem, 2000 
  [14]

- . . . **Kv4.2** mRNA was undetectable.  J Physiol, 2002 
  [13]

- Taken together, these results demonstrate that dendritic, A type K channels are dually regulated by PKA and PKC through a common downstream pathway involving MAPK, and the modulation of these K channels may be accounted for by the phosphorylation of **Kv4.2** subunits.  J Neurosci, 2002 
  [11]

- Since **Kv4.2** channels are involved in protection against back propagating action potentials in dendritic spines, a differential expression of either splice variant could shape the dendritic function.  Mol Cell Neurosci, 2003 
  [10]

- We propose that these functional effects occur as a consequence of the previously reported developmental Kv1.4 to **Kv4.2** Kv4.3 isoform switch.  J Physiol, 1997 
  [10]

- Second, as phosphorylation may regulate channel biophysical properties, differential phosphorylation of **Kv4.2** in the dendrites of pyramidal neurons may confer unique biophysical properties upon particular dendritic input layers.  Learn MemLearn Mem, 1997 
  [10]

- However, they differ from mammalian preparations in which Shal like **Kv4.2** rather than Shaker like Kv1.4 co localize postsynaptically with glutamate receptors.  J Neurocytol, 1999 
  [10]

- DiC8 prevented the augmentation of **Kv4.2** density by quinapril.  Am J Physiol Heart Circ Physiol, 2003 
  [10]

- Therefore, these findings indicate a surprising complexity to kinase regulation of A currents, in that an interaction of two separate molecular events, alpha subunit phosphorylation and the association of an ancillary subunit KChIP3, are necessary for phosphorylation dependent regulation of **Kv4.2** encoded A channels by PKA.  J Neurosci, 2002 
  [7]

- This expression selectively decreased basal phosphorylation of a membrane associated pool of p42 44MAPK, impaired cAMP dependent LTP in the hippocampal Schaffer collateral pathway induced by either forskolin or theta frequency stimulation, decreased complex spike firing, and reduced the p42 44MAPK mediated phosphorylation of the A type potassium channel **Kv4.2**.  Neuron, 2003 
  [7]

- Thus **Kv4.2** and Kv4.3 proteins are important subunits of native rat ventricular I to, and PKC appears to reduce this current by affecting the molecular components of the channels mediating I to .  Am J Physiol, 1997 
  [7]
